# Supplementary material for: Real-Time PCR-Based Screening for Homozygous SMN2 Deletion Using Residual Dried Blood Spots
Source: Genes (Basel). 2023 Nov 29;14(12):2159. doi: 10.3390/genes14122159 (PMC10742981; doi:10.3390/genes14122159)
Supplement: Supplementary file 1 [file genes-14-02159-s001.zip › genes-2736988-supplementary.pdf]

Table S1 Experimental conditions for PCR-RFLP

Table S1-1. Conventional PCR

Table S1-1-1. Conventional PCR reaction mixture

Components	Volume (μL)
Distilled water	10.5
2×PCR Buffer for KOD FX Neo	25.0
KOD FX Neo (1 U/μl)	1.0
dNTPs mixture (A, C, G, T each 2 mM)	10.0
R111 (8 pmol/mL)	1.5
X7-Dra (8 pmol/mL)	1.5
DBS circle (1.2 mm in diameter)	1 piece
Total	50.0

Table S1-1-2. Conventional PCR conditions

	Step	Temperature	Time
1	Initial denaturation	94℃	7 min
2	Denaturation	94℃	1 min
3	Annealing	56℃	1 min
4	Elongation	72℃	1 min
	(30 cycles of steps 2 to 4)		
5	Final elongation	72℃	7 min
6	Hold or storage	15℃	∞

Table S1-2. Restriction enzyme digestion

Table S1-2-1. Restriction enzyme mixture

Components	Volume (μL)
Distilled water	6.0
10×M Buffer	3.0
Dra Ⅰ (15 U/μl)	3.0
PCR products	18.0
Total	30.0

Table S1-2-2. Restriction enzyme treatment conditions

	Step	Temperature	Time
1	Restriction enzyme treatment	37℃	12 hrs
2	Hold or storage	15℃	∞

Table S2 Experimental conditions for real-time PCR

Table S2-1. Real-time PCR reaction mixture

Components	Volume (μL)
Distilled water	3.0
2×PCR Buffer for KOD FX Neo	12.5
KOD FX Neo (1 U/μl)	0.5
dNTPs mixture (A, C, G, T each 2 mM)	5.0
cenSMNex7forw (10 pM)	0.25
cenSMNint7rev (10 pM)	0.25
20x EvaGreen®	2.5
ROX reference dye	0.5
DBS circle (1.2 mm in diameter)	1 piece
Total	25.0

Table S2-2. Real-time PCR conditions

	Step	Temperature	Time
1	Initial denaturation	94℃	7 min
2	Denaturation	94℃	1 min
3	Annealing	56, 58, 60, 62℃	1 min
4	Elongation	72℃	1 min
	(45, 55 cycles of steps 2 to 4)		
5	Final elongation	72℃	7 min
6	Storage	20℃	∞

Table S3. Characteristics of the healthy control

RBC (x10^6/μL)	4.55
Hemoglobin (g/dL)	14.3
Hematocrit (%)	42.0
WBC (/μL)	4700

Table S4. Characteristics of artificial blood samples

Table S4-1. Different hematocrits

	Ht 40%	Ht 60%	Ht 80%
RBC (x10^6/μL)	4.55	7.30	9.63
Hemoglobin (g/dL)	14.3	22.7	29.0
Hematocrit (%)	42.0	66.3	84.7
WBC (/μL)	4700	4800	3200
Estimated DNA amount in a DBS circle (ng)	~70	~70	~50

Table S4-2. Different WBC counts

	Normal WBC count	Low WBC count	Very low WBC count
RBC (x10^6/μL)	5.93	6.33	6.21
Hemoglobin (g/dL)	18.5	20.0	19.3
Hematocrit (%)	53.2	56.7	55.1
WBC (/μL)	4100	700	300
Estimated DNA amount in a DBS circle (ng)	~60	~10	~5

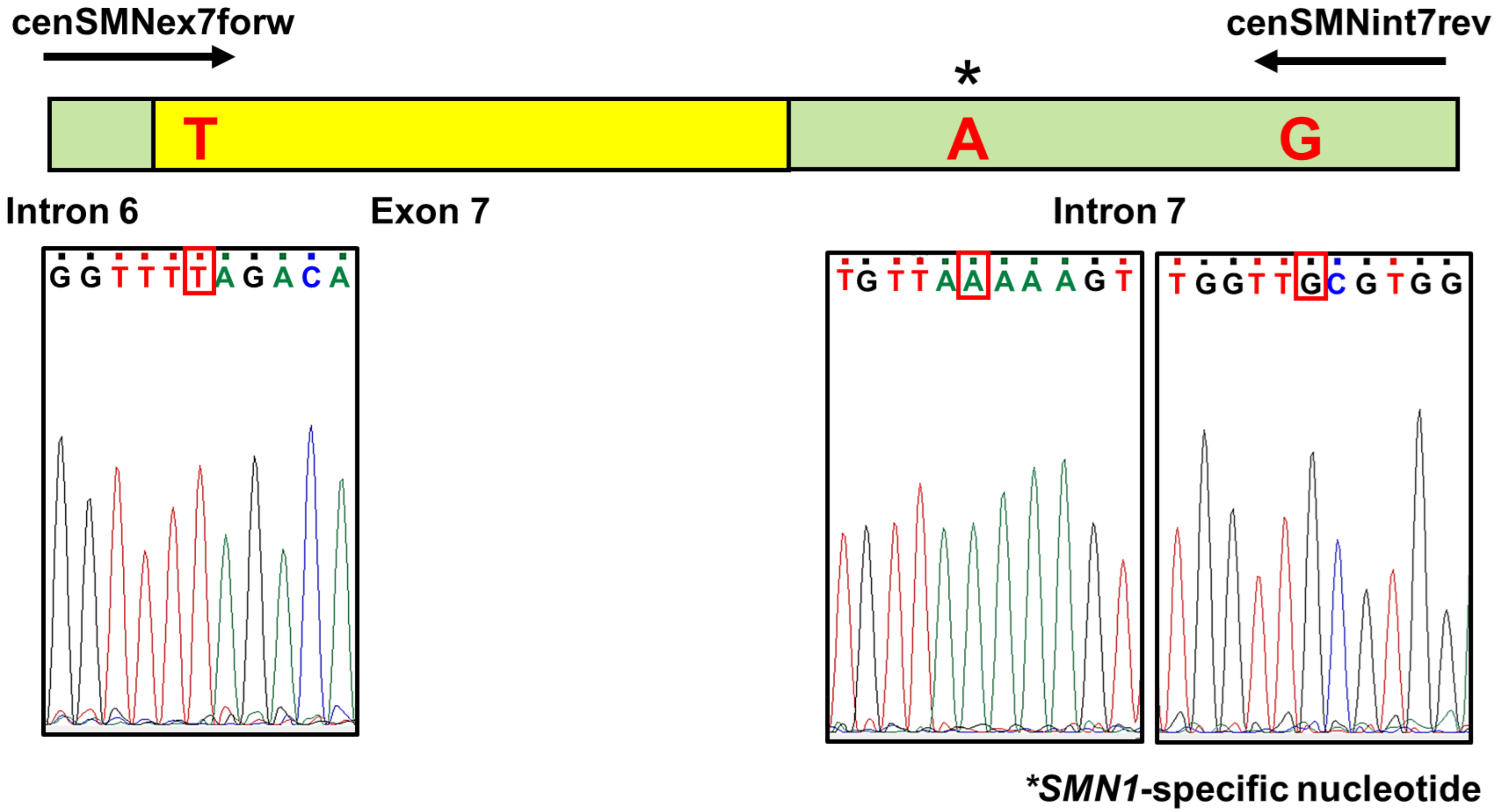

Figure S1. Sequencing analysis of amplified PCR products from DBS with *SMN2* deletion. PCR product from DBS with *SMN2* deletion showed the presence of an *SMN1*-specific nucleotide.

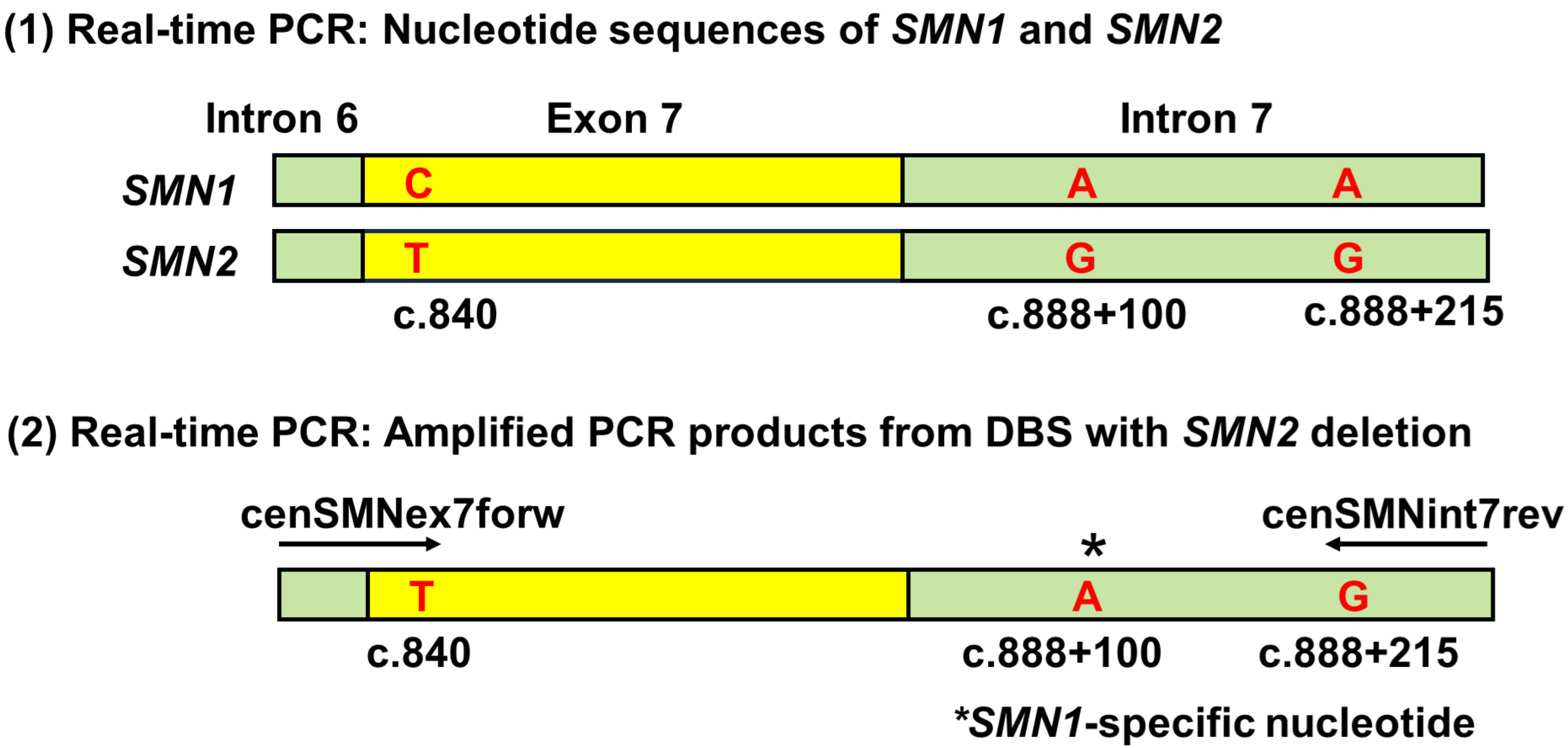

Figure S2. An *SMN1*-specific nucleotide identified in false-negative PCR products. *SMN2*-specific primers can amplify *SMN1* sequences by mis-annealing of the primers to *SMN1* sequence under some conditions including low annealing temperature.
